# Use of the Teach-Back Method in Adults with Cardiovascular Disease: A Scoping Review

**DOI:** 10.3390/healthcare14142093

**Published:** 2026-07-13

**Authors:** Debora Rosa, Elisa Nardin, Sara Aristolao, Richard Sucapuca Ccana, Andrea Poliani, Duilio Fiorenzo Manara

**Affiliations:** 1 Center for Nursing Research and Innovation, Faculty of Medicine and Surgery, Vita-Salute San Raffaele University, 20132 Milano, Italy; rosa.debora@unisr.it (D.R.); sucapuca.richard@hsr.it (R.S.C.); manara.duilio@hsr.it (D.F.M.); 2 Department of Cardiovascular, Neural and Metabolic Sciences, Istituto Auxologico Italiano IRCCS, 20149 Milano, Italy; e.nardin@auxologico.it; 3 ASST Azienda Ospedaliera Papa Giovanni XXIII, 24127 Bergamo, Italy; sara.aristolao@gmail.com; 4 IRCCS Ospedale San Raffaele, 20132 Milano, Italy; 5 Department of Biomedicine and Prevention, Faculty of Medicine, University of Rome “Tor Vergata”, 00133 Rome, Italy

**Keywords:** health education, cardiovascular disease, Teach-Back communication, cardiovascular nursing, self-care, scoping review

## Abstract

**Highlights:**

**What are the main findings?**

- The Teach-Back method has mainly been applied in adults with heart failure, most often during discharge education, early post-discharge follow-up, and transitional care;
- The most consistent improvements were reported in patient knowledge, understanding, self-care behaviors, and treatment adherence; findings on readmissions, healthcare utilization, and other clinical outcomes were less consistent.

**What are the implications of the main findings?**

- Teach-Back should be considered a practical communication strategy for cardiovascular nurses to verify patient understanding and support self-management during high-risk care transitions;
- Future research should standardize Teach-Back delivery, provider training, fidelity monitoring, follow-up timing, and outcome measures to clarify its independent contribution to cardiovascular care.

**Abstract:**

**Background**: Teach-Back is a structured communication strategy increasingly used in patient education to enhance understanding and self-management, particularly among people with complex chronic conditions such as cardiovascular disease. **Aim**: To explore and map how Teach-Back has been applied in adults with cardiovascular disease and to describe outcomes assessed in the literature. **Methods**: A scoping review was conducted following the JBI Manual for Evidence Synthesis and the PRISMA-ScR checklist. MEDLINE/PubMed, CINAHL, Embase, and Scopus databases were searched up to January 2026. Studies involving adult patients with cardiovascular disease receiving Teach-Back–based interventions were included. Data were charted and synthesized descriptively, focusing on intervention characteristics, delivery contexts, and reported outcomes. **Results**: Eighteen studies were included, with heart failure being the most frequently investigated condition. Teach-Back was delivered across hospital, outpatient, and home-care settings, often as part of multicomponent educational or transitional-care interventions. Outcomes were grouped into five domains: knowledge and understanding, self-care and adherence, clinical outcomes, healthcare utilization, and patient-reported outcomes. Improvements were most consistently reported for knowledge, understanding, self-care, and adherence, whereas findings on readmissions and other clinical outcomes were less homogenous. **Conclusions**: Teach-Back appears particularly relevant for cardiovascular nursing practice at points of high-risk communication, including discharge, early post-discharge follow-up, and self-management reinforcement. As a low-resource strategy, it may support nurses in verifying patient understanding and reducing misinterpretation during care transitions. Future studies should standardize delivery, training, fidelity monitoring, documentation, and outcome measurement.

## 1. Introduction

Cardiovascular diseases (CVDs) are the leading cause of death globally and one of the most significant challenges facing healthcare systems. According to the latest estimates from the World Health Organization, approximately 19.8 million people died from cardiovascular causes in 2022, accounting for approximately 32% of all global deaths [1]. In 2021, there were over 600 million prevalent cases of CVD worldwide, with an age-standardized rate of over 7000 cases per 100,000 population [2]. Recent analyses indicate that the global burden of CVD is increasing, in terms of both prevalence and mortality, due in part to aging populations and the spread of modifiable risk factors [2,3,4].

The impact of CVD is not limited to mortality. It results in a significant loss of healthy life years, and contributes substantially to global disability and impaired everyday functioning, including a reduced capacity for physical activity, work and employment, and independent daily living [2,3,4]. Beyond mortality, cardiovascular disease is associated with substantial disability and impaired everyday functioning (for example, a reduced capacity for physical activity, work and employment, and independent daily living) [2,3,4]. In addition to their clinical burden, CVDs have a significant economic impact; in Europe, the estimated total cost exceeds €280 billion per year, more than half of which is attributable to healthcare and long-term care [5].

Effective management of CVD requires a high level of active patient involvement, particularly in terms of treatment adherence, symptom monitoring, diet and fluid intake management, weight control, and lifestyle changes. However, there is considerable evidence that a significant proportion of patients do not fully understand the information they receive during hospitalization and discharge [6,7,8]. The transition from hospital to home is a critical moment in the care pathway, during which communication errors, information overload, and poor health literacy can result in reduced medication adherence, inadequate symptom management, and early returns to the hospital [9,10,11].

In this context, patient education plays a central role in cardiovascular nursing practices. Nurses are frequently involved in planning and delivering educational interventions aimed at supporting patient self-management and empowerment. Tailored, health literacy-sensitive educational approaches have been proposed to encourage greater patient involvement in their care pathways [6,9,10,11]. However, the effectiveness of education depends on the content, the communication methods, and the ability to verify the patient’s understanding. Within cardiovascular care, these educational responsibilities are increasingly positioned within multidisciplinary, team-based models. In such models, advanced-practice and specialist nurses make a substantial contribution to structured patient education, discharge planning, transitional care, and long-term follow-up, particularly for chronic conditions such as heart failure [12,13,14]. Within these multidisciplinary frameworks, the quality of nurse–patient communication is a determinant of whether educational interventions translate into improved self-management.

Among the educational methods used in healthcare, Teach-Back has gradually been established as an effective structured communication strategy. This technique requires the patient to rephrase the information received in their own words, allowing the professional to assess their understanding and intervene in case of misunderstandings [8,15,16]. Teach-Back is a dynamic and iterative process that continues until the patient demonstrates a clear and accurate understanding of the content received [15,16]. It is not a test of the patient’s skills, but a tool for improving the quality of clinical communication [7,15,16,17].

Available evidence suggests that Teach-Back may support the understanding of health information and adherence to treatment plans, although findings vary across settings, populations, and intervention formats [8,15,16,18]. In the cardiovascular field, White et al. reported an improvement in knowledge retention in patients with heart failure, although this was not accompanied by a significant reduction in 30-day readmissions [19]. Peter et al. reported improved patient understanding and a favorable trend in readmission outcomes after the systematic implementation of Teach-Back as part of the discharge process [20]. In a rural setting, Vesterlund et al. documented a 37% reduction in 30-day readmissions following the introduction of an integrated care plan including Teach-Back [21].

Based on this evidence, the application of Teach-Back has been extended to other cardiovascular conditions. In subjects with coronary artery disease, Saadatian et al. reported an improvement in illness perception and self-efficacy [22]. In an outpatient setting, Voelliger et al. used Teach-Back to educate patients to recognize the signs of atrial fibrillation, highlighting the importance of reinforcement interventions over time [23].

Although Teach-Back has been adopted more widely in cardiovascular education, its application across the field remains heterogeneous [8,15,16]. The available literature suggests that Teach-Back is primarily used as a brief communication strategy, as part of discharge teaching, and as one component of broader disease management or care transition interventions. This variability makes it difficult to understand how the method is implemented in cardiovascular care and which outcomes have been assessed most consistently across studies [8,15,16].

This scoping review, therefore, aimed to map how the Teach-Back method has been implemented among adult patients with CVDs, and to describe the range of outcomes assessed across studies. Specifically, the primary objective was to describe how Teach-Back has been implemented in this population, covering its components, delivery format, timing, setting, target cardiovascular conditions, and providers. The secondary objective was to describe the range of outcomes assessed across the included studies and the instruments used to measure them.

## 2. Materials and Methods

This scoping review aimed to systematically map the use of Teach-Back in CVD patient education and the outcomes assessed across care settings. A scoping approach was considered most appropriate as the available evidence was both heterogeneous and conceptually and methodologically fragmented. The literature included different cardiovascular populations, varied settings and providers, diverse intervention components and intensities, and highly variable outcome domains and measurement tools. In addition, Teach-Back was frequently embedded within broader educational or transitional care interventions. This limited the feasibility of direct comparison and made an effectiveness-focused review less suitable at this stage of the evidence base.

This review was developed in accordance with the Joanna Briggs Institute (JBI) Manual for Evidence Synthesis guidelines [24] and reported according to the PRISMA-ScR checklist [25] to ensure transparency, methodological consistency, and traceability of the review process. A protocol was developed a priori to guide the conduct of this scoping review, and was registered on the Open Science Framework platform [DOI: 10.17605/OSF.IO/5C9MX]. The protocol was formulated in accordance with the JBI methodological guidance for scoping reviews [24] to enhance methodological quality, transparency, and consistency throughout the review process. Before starting the study, PROSPERO and the JBI Review Register were consulted to verify the absence of previously published or ongoing protocols on the topic, from which no overlapping studies were identified. The completed PRISMA-ScR checklist, indicating the location of each reporting item within the manuscript, is provided as Supplementary Materials.

The following general research question was stated: “How has Teach-Back been used in adults with CVDs, and in relation to which outcomes?” The question was then divided into three specific sub-questions: (1) How has Teach-Back been used, in terms of its components, duration, timing, and setting? (2) Which cardiovascular populations and which stages of the care pathway were involved in the included studies? (3) Which clinical, behavioral, cognitive, and patient-reported outcomes were evaluated and over what time frames?

In line with the JBI recommendations for scoping reviews [24], the question was structured according to the Population–Concept–Context (PCC) framework (Table 1). In this review, the Population involved adults with CVD; the Concept was Teach-Back as an educational or communication strategy; and the Context included hospitals, outpatient clinics, home settings, and community-based cardiovascular care.

### 2.1. Eligibility Criteria

#### 2.1.1. Inclusion Criteria

Study selection was guided by the research question and the PCC framework [24]. Studies were included if they involved adult patients (≥18 years) with CVD who received a Teach-Back-based educational intervention. Permissible contexts were the hospital, the home environment, and the community. This scoping review included primary sources (randomized and non-randomized trials, observational studies, quasi-experimental studies, and quality improvement projects).

#### 2.1.2. Exclusion Criteria

Studies focusing exclusively on pediatric populations, non-cardiovascular conditions, or educational interventions not involving Teach-Back were excluded. Gray literature, conference abstracts, editorials, and commentaries were also excluded due to limited methodological detail, lack of peer review, and difficulties in evaluating methodological quality.

#### 2.1.3. Language Restrictions and Time Limits

Only studies published in English or Italian were considered. This restriction was to ensure accurate and consistent charting of Teach-Back characteristics and outcome measures. No date restrictions were applied to ensure the full body of evidence on the use of Teach-Back in cardiovascular care was captured, including its earliest applications. The method has been formally adopted in this field only relatively recently, and the number of primary studies remains limited; restricting the time frame would, therefore, have risked excluding potentially relevant evidence.

### 2.2. Search Strategies

As suggested by the JBI guidelines [24], the search strategy consisted of three steps. In the first step, an initial search was performed in MEDLINE using PubMed and the Cumulative Index to Nursing and Allied Health Literature (CINAHL) to identify recurring free terms and the most common Medical Subject Headings (MeSH) controlled terms used in studies related to teach-back. These terms were then combined using Boolean operators (AND/OR). The search strings included terms related to CVDs and the teach-back method: (“Cardiovascular Diseases” [Mesh] OR “Heart Failure” [ Mesh] OR “Coronary Artery Disease” [Mesh] OR “Acute Coronary Syndrome” [Mesh] OR “Myocardial Infarction” [Mesh] OR “Atrial Fibrillation” [Mesh] OR cardiovascular [Title/Abstract] OR “cardiovascular disease*” [Title/Abstract] OR “cardiac patient*” [Title/Abstract] OR “heart failure” [Title/Abstract] OR “coronary artery disease” [Title/Abstract] OR “acute coronary syndrome” [Title/Abstract] OR “myocardial infarction” [Title/Abstract] OR “atrial fibrillation” [Title/Abstract]) AND (“Teach-Back Communication” [Mesh] OR “teach-back” [Title/Abstract] OR “teach back” [Title/Abstract]) (Table S1: Search Strategy). This allowed an analysis of text words and index terms used in the articles. A second search was then carried out in all included databases using the identified keywords to find articles relevant to the research question. In the final stage, the reference lists of eligible articles were searched manually to identify further studies relevant to cardiovascular populations and Teach-Back applications not captured by the primary database search terms. This process enabled the inclusion of studies addressing cardiovascular conditions that were not explicitly listed in the initial search string.

The search was conducted in the MEDLINE (PubMed), CINAHL, Embase, and Scopus databases between 26 and 30 January 2026, with the support of the university library service. When a full text was not available through the indexing platforms, it was requested through the library’s document delivery services.

### 2.3. Document Selection

The selection process was documented using the PRISMA flow diagram 2020 [26]. All identified records were imported into Zotero (v8.0.3) for bibliographic management and deduplication.

Screening was carried out in two stages, managed by the Rayyan web application [27]. In the first stage, two independent reviewers (S.A., E.N.) evaluated titles and abstracts based on eligibility criteria. In the second stage, the same reviewers examined the full texts of potentially relevant articles, recording the reasons for exclusion in a traceable manner. Any discrepancies were resolved through discussion between the reviewers; when necessary, a third senior reviewer (D.R.) was involved in the final decision.

### 2.4. Methodological Quality

Two independent reviewers (S.A. and E.N.) assessed methodological quality using the “Dixon-Woods prompts for assessing quality in primary research” tool [28]. This quality assessment tool was chosen because of anticipated variation in study designs. This tool enabled a consistent appraisal across heterogeneous study designs. Any disagreements between the reviewers were resolved through discussion or with a third senior reviewer (D.R.).

This review involved methodological assessment to provide contextual information on the quality of reporting across the included studies and inform the interpretation of findings. This assessment did not influence the inclusion of studies or the drawing of comparative conclusions. Each included study was appraised using the ten prompts proposed by Dixon-Woods et al. For each prompt, judgments were categorized as “Yes” when the criterion was clearly addressed, “Unclear” when reporting was insufficient to support a definitive judgment, or “No” when the criterion was not met. All prompts were considered equally relevant. One point was assigned for each “Yes” response, and no points for “Unclear” or “No” responses. For each study, the overall percentage was calculated by dividing the number of prompts rated as “Yes” by the total number of prompts and multiplying by 100. The full appraisal is provided in Supplementary Materials (Table S2. Quality appraisal (Dixon-Woods et al., 2005 [28])).

### 2.5. Data Extraction Tool

Zotero (v8.0.3) was used to extract metadata, which was manually checked during import. A standardized tool was used to extract data from the included articles, in accordance with the JBI “Manual for Evidence Synthesis” [24]. Two reviewers (S.A. and E.N.) read and extracted data from the included studies using the data extraction form, completing it in full. The tool recorded details of the evidence source, including the title, authors, year of publication, country, study design, and objectives. It also reported the sample population, specifying age and cardiovascular conditions, as well as describing the Teach-Back intervention, specifying when and by whom it was conducted, and whether a follow-up was included. It also summarized the outcomes analyzed by the studies and the data collection instruments used. The extracted data were synthesized descriptively, combining a narrative approach with basic frequency counts and tabulation. The reported outcomes were grouped into conceptually coherent domains, and the findings were summarized in terms of the direction and consistency of the results reported by the primary studies, without pooling data or estimating effect sizes.

## 3. Results

A total of 311 records were identified through database searches. After removing duplicates, the number of articles was reduced to 276. Screening of titles and abstracts led to the exclusion of 229 studies (83%) irrelevant to the research question, mainly due to the absence of a Teach-Back intervention or non-CVD populations. Forty-seven articles remained for full-text evaluation.

**Figure 1:**
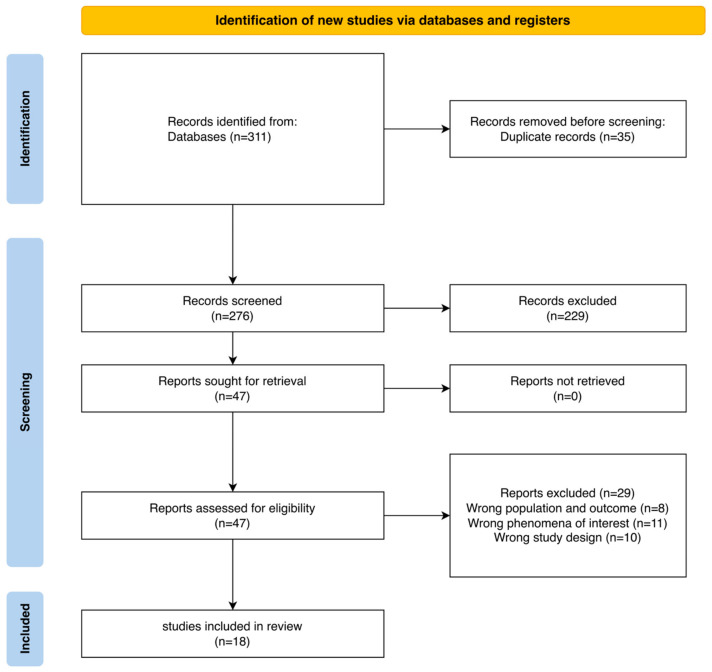
PRISMA flow diagram 2020 [26].

During the full-text review phase, an additional 29 studies (61.8%) were excluded for failing to meet eligibility criteria, primarily due to insufficient description of the intervention, absence of outcomes consistent with the objective of the review, or use of Teach-Back that was not clearly defined. Following this stage, a total of 18 studies (N = 18) met all eligibility criteria and were included in the review, as reported in the PRISMA 2020 flow diagram (Figure 1).

### 3.1. Characteristics of the Included Studies

The included studies were published between 2013 [19] and 2023 [29]. Study design comprised five randomized controlled trials [29,30,31,32,33], seven quasi-experimental or controlled comparative studies [22,34,35,36,37,38,39], four quality improvement projects [20,21,40,41], one observational study [19], and one prospective study [23]. The article’s characteristics are presented in Table 2; Teach-Back interventions, outcomes, and key findings of each study are summarized in Table 3.

**Figure 2:**
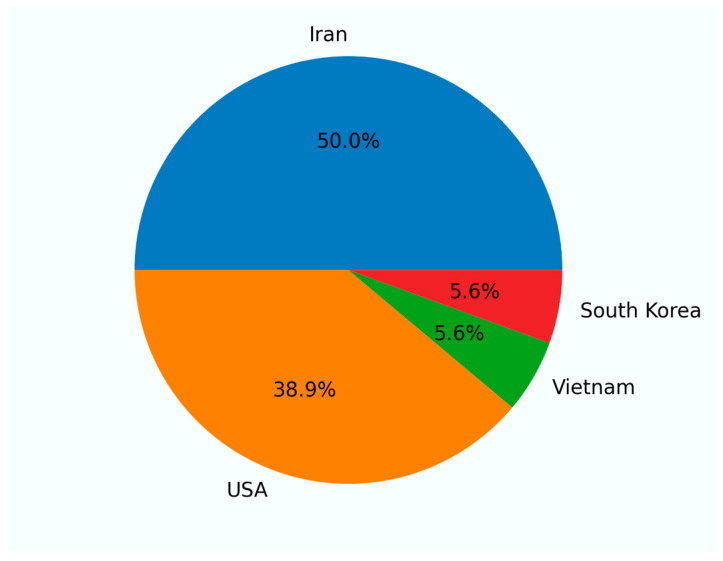
Countries where the studies were conducted.

Figure 2 shows the geographical distribution of the included studies. The available evidence on Teach-Back in the education of patients with cardiovascular disease was limited to North America [19,23,35,38,39,40,41] and parts of Asia and the Middle East, including Vietnam, South Korea, and Iran [20,21,22,29,30,32,33,34,36,37]. The publication years of the included studies ranged from 2013 [19] to 2023 [29].

### 3.2. Quality Appraisal

A methodological assessment of the quality of reporting across the included studies was conducted to enable more accurate interpretation of the findings. The appraisal was used to contextualize the evidence without influencing the inclusion of studies or serving as a basis for comparative evaluation.

Given the heterogeneity of the included study designs, methodological quality was assessed using the Dixon-Woods Prompts for Assessing Quality in Primary Research tool [28]. The included studies showed moderate-to-high methodological quality, with scores ranging from 70% to 100%. The main weaknesses concerned incomplete reporting of intervention delivery, provider preparation, and fidelity to the Teach-Back method, rather than major flaws in study intent or outcome reporting. (Supplementary Table S2).

### 3.3. Cardiovascular Conditions

Table 4 shows the cardiovascular conditions represented in the included studies. The largest group of studies focused on heart failure (HF). Specifically, 13 studies (13/18; 72.2%) enrolled patients with HF, including both hospitalized patients and those at follow-up after discharge [19,20,21,29,30,31,32,34,35,37,38,41]. In several of these studies, HF was reported without further specification of severity or functional class [30,33,34,35,41]. Other studies defined the sample more precisely according to the New York Heart Association (NYHA) classification system, including NYHA I–IV [32] and NYHA II–IV [31,37] patients. Oh et al. [29] included patients with HF and a left ventricular ejection fraction of less than 50%, whereas Rahmani et al. [38] focused on patients with advanced HF and an ejection fraction of 40% or less. Vesterlund et al. [21] enrolled patients admitted with primary HF.

The remaining studies addressed other cardiovascular conditions. Azizi et al. investigated patients with acute coronary syndrome [36], while Saadatian et al. included patients with coronary artery disease [22]. Voelliger et al. focused on patients receiving education on radial pulse self-palpation and symptom recognition for atrial fibrillation detection [23]. Bates et al. examined patients who had undergone coronary artery bypass grafting (CABG) [40]. Zabolypour et al. enrolled patients with hypertension and evaluated adherence to treatment regimens following Teach-Back education [39]. Overall, the evidence base was heavily weighted towards HF, while other cardiovascular conditions were represented by single or small clusters of studies.

### 3.4. Age

Age was one of the eligibility criteria considered in the included studies but was reported inconsistently. Several studies explicitly required participants to be adults aged 18 years or over [19,23,31,32,35]. In other cases, the inclusion criteria were more restrictive, focusing on specific adult subgroups, such as individuals aged 30 years or over [37], 65 years or over [39], or within predefined age ranges of 45–85 years [22] and 18–70 years [21]. Some studies explicitly targeted older adult populations, particularly those aged over 65 [38]. Other included studies also enrolled adult participants, but the age thresholds were variably reported or not described in enough detail to allow consistent categorization across all studies [20,29,30,33,34,36,40]. Only one study did not clearly report the age criterion for participant inclusion [41].

### 3.5. Intervention Description

The duration and mode of delivery of the Teach-Back intervention varied considerably across the included studies. In some cases, it was delivered face-to-face; for instance, Karami Salaheddin Kola et al. (2021) described an intensive, consecutive, four-day program comprising daily sessions lasting 20–30 min for patients [32]. Other studies included single or multiple educational sessions ranging from 15 to 60 min in duration [20,21,35,37]. White White et al. (2013) reported an average session length of 34 min (range 15–120 min) [19]. Some interventions consisted of initial educational meetings and subsequent follow-up contacts [19,20,22,23,31,32,35,37,39,40,41]. The average length of the educational session was not specified in some studies [22,23,31,38,40,41]. Others used the Teach-Back method as part of wider structured educational interventions. However, the available reports provided inconsistent details regarding session duration, delivery format, and follow-up procedures [29,30,33,34,36].

Teach-Back sessions were often delivered during hospitalization [20,23,32,35,37,39,41]. In some studies, the timing was specified more precisely, for example, as over four consecutive days [32], on the day of discharge [20], or before discharge [22,31]. In other cases, the intervention was described in relation to the clinical pathway, for instance, from day 1 to day 3 plus the day before discharge [38], or during the post-CABG phase [40].

Not all interventions were limited to the hospital or outpatient clinic setting. Saadatian et al. (2022) scheduled three Teach-Back sessions over three days: the first at the patient’s bedside, the second one day after admission, and the third at the patient’s home [22].

Follow-up was primarily conducted by telephone, at various time points ranging from 48 to 72 h after discharge [40] to one week [19,29,41], two weeks [32], three weeks [23], one month [20], three months [31,37,38], or ninety days [35] after intervention. Some studies also included in-person follow-up visits with a general practitioner or cardiologist a few days after discharge [21,41]. Saadatian et al. (2022) did not report any follow-up specifically related to the Teach-Back method [22]. In one study, follow-up focused on hospital readmission rather than the Teach-Back content itself [38].

### 3.6. Providers of Teach-Back

As shown in Table 5, the Teach-Back method was predominantly delivered by nurses or nursing staff across the included studies. In 12 studies, the provider was explicitly identified as a nurse, a nursing team, a registered nurse, or clinical nursing staff [19,20,21,22,23,29,31,35,36,37,38,41]. This pattern was particularly evident in HF settings, where education was commonly integrated into routine nursing care or discharge preparation. In two additional studies, the intervention appeared to have been delivered in a nursing context, although the provider was not clearly specified [30,34]. In contrast, the intervention was delivered in one study by a dedicated patient educator [40] and in another by a researcher, rather than clinical staff [33]. In the remaining studies, the identity of the provider was either only partially described or not reported at all [32,39].

The data in Table 5 also illustrate that reporting on Teach-Back training was limited and inconsistent. Only five studies clearly described a structured form of provider preparation: Awoke et al. reported three consecutive ‘lunch-and-learn’ sessions for nurses [35]; Bates et al. described preparation based on the Health Literacy and Patient Safety clinician manual [40]; Peter et al. reported a 20 min eLearning module plus a mandatory two-hour train-the-trainer workshop [20]; Vesterlund et al. described PowerPoint-based education with pre/post knowledge testing [21]; and White et al. reported training through an Institute for Healthcare Improvement course [19]. Two further studies referred to provider preparation in more general terms, but without sufficient detail to assess its content or intensity: Dinh et al. simply stated that nurses had received Teach-Back training [31], while Oh et al. described the intervention nurse as being well trained and experienced in cardiovascular care, without specifying the training pathway [29].

### 3.7. Outcomes

#### 3.7.1. Clinical Outcomes: Readmissions, Resource Utilization, and Length of Stay

The most frequently reported clinical outcome was 30-day readmission, particularly in HF and post-discharge care studies [19,20,21,37,40,41]. Evidence in this area mainly came from quality improvement initiatives and observational studies, with fewer data from randomized trials. In a rural HF quality improvement project, the implementation of an integrated discharge plan that incorporated the Teach-Back method was associated with a 36.9% reduction in readmissions [21]. In a structured, multidisciplinary HF program, the overall 30-day readmission rate decreased from 27.0% to 10.2% during the implementation period (*p* < 0.001) [41]. In patients who had undergone CABG, the incremental implementation of STAAR interventions (STate Action on Avoidable Rehospitalizations) that incorporated the Teach-Back method reduced 30-day readmissions from 25.8% to 12.0% [40]. These findings suggest that the Teach-Back method may contribute to better transitional care when embedded within broader discharge bundles and follow-up pathways [21,40,41].

Results were less consistent when Teach-Back was examined outside of an organizational intervention bundle. In the study by White et al., correct Teach-Back responses during hospitalization were not significantly associated with lower all-cause 30-day readmission (*p* = 0.775); the same was true at follow-up (*p* = 0.609), despite good knowledge retention [19]. In the study by Awoke et al., 30-day readmission did not improve significantly, although knowledge and self-care improved [35]. In the recent randomized trial by Oh et al., unplanned healthcare resource utilization at one month did not differ significantly between groups [29]. Taken together, Teach-Back appears more likely to influence readmissions when embedded within structured care-transition programs. When evaluated independently, its effect on clinical outcomes remains uncertain [19,21,29,35,40,41].

#### 3.7.2. Cognitive Outcomes: Knowledge and Illness Perception

Knowledge was one of the most consistently improved outcomes across the included studies [31,35,38]. In Awoke et al., HF knowledge improved significantly at seven days (*p* ≤ 0.001) and remained higher at 90 days (*p* ≤ 0.032) following a nurse-led Teach-Back program [35]. In Dinh et al., the intervention group demonstrated significantly greater HF knowledge than the usual care group at follow-up, alongside better self-care maintenance [31]. Rahmani et al. also reported significant post-intervention gains in knowledge that persisted at three months [38]. Taken together, these findings suggest that the Teach-Back method is consistently associated with improved acquisition and retention of disease-related information in adults with HF [31,35,38].

Broader cognitive outcomes were also affected. Saadatian et al. found that, in patients with coronary artery disease, illness perception improved significantly after one month (*p* < 0.001) and self-efficacy remained higher in the intervention group (*p* = 0.007) after adjustment for baseline values [22]. This is relevant because it suggests that Teach-Back may influence not only factual knowledge, but also patients’ interpretation of illness and confidence in cardiovascular self-management [22].

#### 3.7.3. Self-Care and Self-Care Behaviors

Self-care was the outcome domain with the most coherent pattern across randomized and quasi-experimental studies [29,30,31,32,37]. Significant improvements in self-care maintenance (F = 11.597; *p* = 0.001), symptom perception (F = 20.173; *p* < 0.001), self-care management (F = 7.205; *p* = 0.009), and self-care efficacy (F = 4.210; *p* = 0.043) were reported following Teach-Back-based discharge education at 7 days in the randomized trial by Oh et al. [29]. In the study by Dinh et al., the intervention group showed significantly better self-care maintenance than the usual care group over the follow-up period [31]. These data indicate an early effect of Teach-Back on patients’ ability to manage symptoms after discharge [29,31].

Quasi-experimental studies showed a similar direction of effect. In the study by Dalir et al., self-care behavior improved significantly at 1 month post-intervention (*p* < 0.001), whereas no significant change was observed in the control group (*p* = 0.138) [30]. Awoke et al. reported that self-care maintenance improved at seven and 30 days (both *p* ≤ 0.001), self-care management improved at seven (*p* ≤ 0.001) and 30 days (*p* = 0.013), and self-care confidence improved at 30 days (*p* = 0.017) [35]. Mesbahi et al. likewise reported significantly better self-care behavior at three months (*p* < 0.001), together with fewer readmissions (*p* = 0.002) [37]. In the three-arm trial by Karami et al., all educational approaches improved self-care; between-group differences were limited, with a significant advantage observed just before discharge (*p* = 0.04), while blended learning performed better for certain secondary outcomes [32].

Together, the included studies consistently reported improvements in self-care following Teach-Back–based education, particularly during the early post-discharge period. However, because Teach-Back was frequently delivered alongside other educational components, these findings describe an overall influence rather than demonstrating an independent effect [29,30,31,32,35,37].

#### 3.7.4. Treatment Adherence, Quality of Life, and Patient-Reported Outcomes

Patient-reported outcomes were assessed less frequently than knowledge or self-care, but the findings were generally favorable [23,33,34,36,38,39]. In patients with acute coronary syndrome, Azizi et al. found that treatment adherence increased from 56.22 ± 12.98 at one month to 61.22 ± 11.51 at three months in the intervention group (*p* < 0.05); in the same period, adherence worsened in the control group (*p* = 0.04) [36]. In HF patients, Aghamohammadi et al. reported that medication adherence was significantly higher in both active education groups than in the control group immediately after training (*p* < 0.001). At six weeks, the Teach-Back group performed better than both the control and visual education groups (*p* < 0.001) [34]. In a sample of patients with CVD, Zabolypour et al. found that the Teach-Back method improved adherence to the therapeutic regimen at two months, although motivational interviewing produced a larger improvement [39]. These findings suggest that the Teach-Back method can support adherence, but the extent of the benefit may vary depending on the condition, the comparator, and the follow-up period [34,36,39].

When assessed as an outcome, quality of life was also shown to improve. In the study by Rahmani et al., Teach-Back was associated with a significant improvement in quality of life after three months, with improvements observed in most SF-36 domains [38]. Mohammadi et al. reported that a multimedia educational program combined with Teach-Back improved quality of life and reduced cardiac anxiety immediately after the intervention and at one and three months (all *p* < 0.05) [33]. Teach-Back was also employed to promote symptom recognition and self-monitoring; in Voelliger et al., 88% of participants correctly performed radial pulse palpation, and 93% identified at least one symptom of atrial fibrillation immediately after education. At three weeks, 94.7% were still performing daily self-screening; however, only 44.7% could still verbally recall at least one symptom, indicating attrition in symptom recall over time [23]. Overall, these studies suggest that the Teach-Back method may improve adherence, quality of life, and symptom monitoring. However, sustained benefits appear more likely when education is reinforced after discharge [23,33,38].

Across the included studies, the most consistent signal concerned improvements in patients’ understanding of their condition, self-care behaviors, and treatment adherence. In contrast, effects on readmissions and healthcare utilization were more variable and more difficult to attribute specifically to Teach-Back.

### 3.8. Outcome Measures and Data Collection Instruments

As shown in Table 6, the included studies employed a diverse set of data collection methods. These methods are most effectively interpreted by categorizing them as either validated instruments, non-validated measures, or clinical and administrative data sources. The most frequently used validated instruments assessed HF knowledge and self-care. Three studies used the Dutch Heart Failure Knowledge Scale (DHFKS) [31,32,35]. Three used the Self-Care of Heart Failure Index (SCHFI), with version 6.2 used by Awoke et al. and Dinh et al., and version 7.2 used by Oh et al. Three additional HF studies used the European Heart Failure Self-Care Behavior Scale (EHFScBS) [30,32,37]. Other validated instruments were used less frequently and assessed broader outcome domains. These included medication adherence (MMAS-8 in Aghamohammadi et al. [34]; the Scale of Adherence to Systemic Hypertension Treatment in Zabolypour et al. [39]); quality of life and psychological outcomes (SF-36 and 16-Item Cardiac Self-Care Questionnaire in Rahmani et al. [38]; Minnesota Living with Heart Failure Questionnaire and Cardiac Anxiety Questionnaire in Mohammadi et al., [33]); illness perception and self-efficacy (Brief IPQ and CMSES in Saadatian et al. [22]); and perceived social support (MSPSS in Karami, Salaheddin et al., [32]). Additionally, some studies used clinical indices rather than patient-reported outcome measures. Examples include the Charlson Comorbidity Index used in Dinh et al. [31] and Oh et al. [29] and the CHA_2_DS_2_-VASc score in Voelliger et al. [23]. Alongside these validated tools, several studies relied on non-validated or study-specific measures. These included demographic forms, questionnaires, patient experience surveys, confidence and conviction scales, structured Teach-Back knowledge questions, structured documentation of education and call-back questionnaires, and a structured assessment of pulse self-check. Some outcomes were derived from clinical or administrative sources rather than from direct patient questionnaires. These sources included electronic health records, routine clinical and quality improvement data, program audit data, readmission indicators, readmission and visit records, and mortality data from the Social Security Death Index (Table 6).

## 4. Discussion

From the perspective of cardiovascular nursing, the findings of this scoping review suggest that the Teach-Back method is particularly relevant during key educational moments—such as discharge and early post-discharge follow-up—and for self-management reinforcement. These are critical for patients with CVD, especially those with HF, who risk misunderstanding their treatment plans, symptom monitoring, adherence requirements, and daily self-care tasks. The review showed that the Teach-Back method has been implemented in a wide range of cardiovascular educational contexts. Most studies focused on HF due to its high clinical burden, frequent instability, and impact on healthcare utilization. The latter includes hospital readmissions, one of the most frequently investigated outcomes. Across the included studies, favorable trends were mainly reported in knowledge, self-care, adherence, and—less consistently—healthcare utilization.

However, these findings should be interpreted cautiously, because Teach-Back was often embedded within varied, multicomponent interventions, making it difficult to isolate its specific contribution. Indeed, a major finding of this review was the substantial heterogeneity of interventions, which differed in terms of repetition, duration, timing, setting, and follow-up. Educational sessions were delivered as single or repeated encounters, before, during, or after hospitalization. Where follow-up was included, it was scheduled at different time points [19,20,23,35,36,38,39,40,41]. Several studies did not clearly report the duration of educational sessions [22,23,31,38,40,41]. This variability extended to the settings in which Teach-Back was implemented, which included hospitals, outpatient clinics, and patients’ homes. Currently, this variability prevents us from drawing conclusions about the most suitable context for its delivery, its greatest potential impact, or whether its effect may be strengthened when initiated in a hospital and continued across care transitions. Taken together, these differences highlight the absence of shared operational standards for Teach-Back in cardiovascular care and underscore the need for greater standardization in intervention delivery and outcome assessment.

Another important finding was the central role of nurses in delivering Teach-Back [19,20,21,22,23,31,38,39]. This is highly relevant from a cardiovascular nursing perspective because it highlights nurses’ educational and relational responsibilities in chronic disease management and transitional care. Some studies reported that nurses received specific preparation or training before delivering Teach-Back [39], a factor not discussed in many others [19,21,22,23,37,41]. The limited reporting on staff preparation raises an important practical issue: whether effective use of Teach-Back requires formal, structured training or basic orientation alone. Due to differences in nursing education and professional roles across countries, no firm conclusion can be drawn from the available evidence. However, one point is clear: Teach-Back should not be improvised. If clinicians are not confident in their ability to apply the method correctly, the quality and consistency of patient education may be compromised. For this reason, nurse managers and coordinators should promote awareness of the method and verify Teach-Back competence in practice, activating dedicated training when needed.

Follow-up in included studies was often conducted by telephone [19,20,31,32,35,37,38,39,40,41], suggesting the suitability of digital platforms, video calls, or app-based support to reinforce Teach-Back over time. However, this possibility should be approached with caution, as the feasibility of such approaches may vary considerably across healthcare systems and organizations. Furthermore, digital approaches may unintentionally exclude older patients, those with limited digital confidence, and those without access to appropriate devices.

To evaluate outcomes, rather than directly comparing highly heterogeneous measures, the findings were synthesized into five conceptually coherent domains: clinical outcomes [19,21,29,41]; healthcare utilization [19,21,29,41]; cognitive outcomes, including disease-related knowledge, illness perception, and symptom recognition [19,23,31,35,38]; behavioral outcomes, including self-care and treatment adherence [29,30,31,32,34,35,36,37,39]; and patient-reported outcomes, including quality of life, cardiac anxiety, self-efficacy, and perceived social support [22,23,33,38]. This approach was necessary due to marked variability across studies in terms of populations, cardiovascular conditions, intervention characteristics, follow-up timing, and measurement tools. Accordingly, our results should be interpreted as a narrative synthesis describing the direction and pattern of effects, rather than as directly comparable estimates of effectiveness. The outcomes were evaluated using a variety of instruments—including the DHFKS, SCHFI, EHFScBS, SF-36, Brief-IPQ, CMSES, MMAS-8, MLHFQ, and CAQ—with limited overlap between studies. The inconsistency in outcome selection and measurement reduces comparability, supporting the need for a core outcome set for future Teach-Back research in cardiovascular care. This set should be defined through consensus processes involving patients, clinicians, and researchers.

The geographical distribution of the evidence also deserves attention. Most included studies were conducted in North America [19,23,35,38,39,40], some parts of Asia, and the Middle East [20,21,22,29,31,32,33,34,36,37]; no studies were identified from Europe, Latin America, Africa, or other parts of Asia. This limited geographic spread reduces the transferability of the results. This is particularly relevant for an intervention such as Teach-Back, whose success is closely linked to language, communication style, and cultural expectations. Additionally, health literacy was not consistently assessed across studies. This makes it difficult to determine whether interventions were well adapted to patients’ understanding levels or if literacy influenced intervention duration or intensity. Given the diversity of patients’ linguistic and cognitive abilities, it is likely that tailored educational approaches will be essential [9,10,11,15]. Differences in healthcare systems may have also influenced patient selection, intervention delivery, follow-up, and choice of outcomes. However, these contextual factors could not be systematically examined from the available evidence.

Finally, the overall methodological quality of the studies included was variable but acceptable. Some studies had strong designs and clear reporting [20,22,31,32,41], while others provided limited detail on analytical methods, intervention fidelity, or reproducibility [19,21,23,35,37,38,39,40]. Although randomized and quasi-experimental studies strengthen the evidence base, substantial heterogeneity across studies limits direct comparison. Observational studies and quality improvement projects suggest that Teach-Back is already being used in routine practice, often without standardization. This indicates that the quality of educational delivery may depend more on individual professionals than on shared protocols. A clearer, more structured understanding of how Teach-Back is implemented, delivered, and evaluated could support more consistent, evidence-based, and context-sensitive use in cardiovascular care.

### 4.1. Limitations

Several methodological limitations should be considered when interpreting these findings. First, the included evidence was highly heterogeneous in study design—encompassing randomized controlled trials, quasi-experimental and observational studies, and quality improvement projects—as well as in the examined populations, settings, follow-up timing, and outcome measures. This heterogeneity precluded any quantitative synthesis and limits the comparability of findings across studies. Second, in many studies, Teach-Back was not delivered as a discrete intervention but was embedded within multicomponent educational programs, discharge bundles, or transitional-care interventions; the reported outcomes cannot therefore be specifically attributed to the Teach-Back method itself. Third, fidelity to the Teach-Back method was rarely assessed, and reporting of provider background and preparation was inconsistent, ranging from structured training to no description at all. This limits any conclusion about the conditions required for effective delivery. Fourth, the available evidence was geographically concentrated in North America and parts of Asia and the Middle East, with no studies identified from Europe, Latin America, or Africa. Because the effectiveness of Teach-Back is closely linked to language, communication style, and cultural expectations, this concentration could reduce the transferability of the findings. Additionally, the review was limited to studies published in English or Italian. This choice was made to ensure that the included literature could be accurately interpreted, critically assessed, and consistently synthesized by the review team. However, this language restriction may have introduced language bias and led to the exclusion of potentially relevant studies published in other languages. Finally, because the concept block of the search was deliberately built around the specific term “Teach-Back”, a small number of studies describing the technique under different labels may not have been retrieved by the database search, although hand-searching of reference lists was undertaken to mitigate this risk.

### 4.2. Implications for Clinical Practice

This review has several implications for cardiovascular nursing practice. Teach-Back appears particularly suited to high-risk communication points—especially discharge, early post-discharge follow-up, and self-management reinforcement—at which patients are required to understand and apply complex treatment instructions. In these contexts, Teach-Back should be considered a core communication strategy to verify understanding and reduce the risk of misinterpretation during care transitions.

The findings also suggest that Teach-Back is best embedded within routine clinical workflows, rather than delivered as a stand-alone educational intervention. Its application aligns closely with the role of cardiovascular nurses in patient education, discharge planning, and continuity of care, particularly in populations with HF and other chronic cardiovascular conditions that require ongoing self-management. This observation is consistent with international guidance and reviews advocating multidisciplinary, team-based models of cardiovascular care, particularly for HF; structured, nurse-led education and the verification of patient understanding are regarded as core components of effective transitional care [13,14]. Positioning Teach-Back explicitly within such multidisciplinary pathways may help ensure that educational efforts are reinforced across care transitions and shared consistently among the members of the care team, rather than depending on the initiative of individual professionals.

From an implementation perspective, the current evidence highlights the need to move beyond informal or inconsistent use of Teach-Back. Structured adoption should include dedicated staff preparation, explicit incorporation into care pathways, and consistent documentation practices. Training should focus not only on the use of the technique itself, but also on adapting communication to align with patients’ health literacy, cognitive capacity, and clinical condition.

Although evidence on clinical endpoints remains inconsistent, the more robust findings presented here on knowledge, self-care, and adherence are clinically meaningful. In practice, improvements in these domains may contribute to safer care transitions, better patient engagement, and more effective long-term management of CVD.

### 4.3. Implications for Future Research

Future studies should provide clearer information on who delivers Teach-Back, the training they receive, how competence is assessed, and whether fidelity to the method is monitored over time. There should also be greater consistency in the timing of follow-ups and the selection of outcomes; this is particularly important if future research aims to clarify whether Teach-Back has an independent effect on readmissions or healthcare utilization beyond its role in broader, multicomponent interventions. Further research should also examine how Teach-Back is positioned within multidisciplinary and advanced-practice nursing models of cardiovascular care, and define the competencies and educational standards required for nurses to deliver it consistently across settings [12]. The development of a core outcome set—established through consensus processes involving patients, clinicians, and researchers—would additionally improve the comparability of future studies.

## 5. Conclusions

This scoping review shows that the Teach-Back method has been used in various cardiovascular care settings, primarily for HF patients during discharge and early care transitions. The most consistent pattern across the included studies was an improvement in patients’ understanding of their condition, self-care behaviors, and treatment adherence. However, findings on readmissions, healthcare utilization, and other formal clinical outcomes were less consistent and more difficult to attribute specifically to Teach-Back. This distinction is important, as it suggests that Teach-Back’s main contribution to cardiovascular care may be improved communication quality and support for patients’ ability to manage complex treatment demands, rather than producing measurable effects on clinical endpoints.

The descriptive evidence synthesized in this review positions Teach-Back as a clinically relevant and pragmatic communication strategy for cardiovascular nursing practice. It has particular value in care phases where misunderstandings may compromise continuity, safety, and self-management. However, the current evidence base is limited by substantial heterogeneity in populations, settings, intervention design, co-interventions, follow-up timing, and outcome measurement. In many studies, Teach-Back was embedded within broader educational or transitional care programs, preventing firm conclusions about its independent effectiveness.

Therefore, the value of Teach-Back should not be simplistically interpreted as either proven or unproven. Rather, the available literature suggests Teach-Back as a pragmatic communication method with strong clinical rationale and encouraging signs of benefit. However, further rigorous and standardized investigations in cardiovascular populations are required. Future research should clarify how Teach-Back is implemented, who delivers it, what training is required, how fidelity is monitored, and which outcomes should be consistently assessed across studies. A more robust and standardized evidence base would support the wider implementation of Teach-Back in cardiovascular care and strengthen its role in evidence-based nursing practice.

## Figures and Tables

**Table 1:** Population–Concept–Context framework.

**P—Population**	Adults (aged ≥18 years) with cardiovascular diseases (e.g., heart failure, ischemic heart disease, acute coronary syndrome, atrial fibrillation, or hypertension).
**C—Concept**	Teach-back can be used as an educational or communication strategy on its own or as part of a multimodal intervention, for example, alongside discharge bundles, disease management programs, or structured nursing interventions.
**C—Context**	This applies to any healthcare setting, including hospitals (inpatient and outpatient care), home follow-up, and community/local areas. This includes care transition settings, particularly hospital discharge and close follow-up after discharge.

**Table 2:** Characteristics of the included studies (*n* = 18).

Author (Year) Country	Design and Objectives	Population	Setting
Aghamohammadi (2020) [34] Iran	Controlled comparative trial; comparing Teach-Back vs. pictographic education on medication adherence	*n* = 210 HF; adults	Hospital cardiology ward
Awoke (2019) [35] Ethiopia	Quasi-experimental; evaluating a nursing education program on knowledge and self-care	*n* = 80 HF	Cardiology clinic
Azizi (2020) [36] Iran	Quasi-experimental; evaluating the effect of Teach-Back on treatment adherence	*n* = 70 ACS	Coronary care unit
Bates (2014) [40] USA	Quality improvement project; reduction in post-CABG readmissions	*n* = 189 CABG	Tertiary care hospital
Charais (2020) [41] USA	QI program; evaluating HF disease management	*n* ≈ 150 HF	Military medical center
Dalir (2015) [30] Iran	RCT; effect of Teach-Back on HF self-care	*n* = 62 HF	Cardiology ward
Dinh (2019) [31] Vietnam	Cluster RCT; effectiveness of the Teach-Back HF program	*n* = 140 HF	Cardiology wards
Karami (2021) [32] Iran	RCT; Teach-Back vs. multimedia on self-care	*n* ≈ 150 HF	Hospital
Mesbahi (2020) [37] Iran	Quasi-experimental; Teach-Back on self-care and readmissions	*n* = 80 HF	CCU
Mohammadi (2021) [33] Iran	RCT; Teach-Back on QoL and cardiac anxiety	*n* ≈ 120 HF	Hospital
Oh (2023) [29] South Korea	RCT; Teach-Back discharge program	*n* ≈ 100 HF	University hospital
Peter (2015) [20] USA	QI project; reduction in HF readmissions	*n* ≈ 300 HF	Magnet hospital
Rahmani (2020) [38] Iran	Quasi-experimental; Teach-Back on knowledge and QoL	*n* ≈ 70 HF	Cardiology
Saadatian (2022) [22] Iran	Quasi-experimental; Teach-Back on illness perception	*n* = 100 CAD	CCU
Vesterlund (2015) [21] USA	QI HF project	*n* ≈ 120 HF	Rural hospital
Voelliger (2021) [23] Switzerland	Prospective study	*n* ≈ 80 atrial fibrillation	Cardiology clinic
White (2013) [19] USA	Observational study	*n* ≈ 276 HF	Hospital
Zabolypour (2020) [39] Iran	Comparative trial; Teach-Back vs. motivational interviewing	*n* ≈ 90 CVD	Hospital

ACS, acute coronary syndrome; CABG, coronary artery bypass grafting; CAD, coronary artery disease; CCU, coronary care unit; CVD, cardiovascular disease; HF, heart failure; QI, quality improvement; RCT, randomized controlled trial.

**Table 3:** Teach-Back interventions, outcomes, and key findings of the included studies.

Author (Year) Country	Teach-Back Intervention	Follow-Up	Outcomes and Instruments	Key Findings
Aghamohammadi (2020) [34] Iran	3 consecutive educational sessions; explanation + request for the patient to repeat treatment instructions; nurse educator	6 weeks	Medication adherence (MMAS-8)	Teach-Back significantly improved adherence compared to the control
Awoke (2019) [35] Ethiopia	Structured educational program with comprehension assessment via Teach-Back; individual sessions	3 months	HF knowledge (DHFKS), self-care (SCHFI)	Significant increase in knowledge and self-care behaviors
Azizi (2020) [36] Iran	3 sessions (30–45 min); patient rephrases treatment instructions; nurses	1 and 3 months	Treatment adherence	Significant improvement in adherence
Bates (2014) [40] USA	Teach-Back at discharge + scheduling of cardiology follow-up visit; patient educator	30 days	Hospital readmissions; patient experience survey	Significant reduction in readmissions
Charais (2020) [41] USA	Educational bundle with Teach-Back at discharge; multidisciplinary team	30 days	HF readmissions	Reduction in readmission rate
Dalir (2015) [30] Iran	Individual education with patient repetition of content	1 month	HF self-care (EHFScB scale)	Significant improvement in self-care
Dinh (2019) [31] Vietnam	Education session + written materials + Teach-Back assessment; nurses	1 and 3 months	HF knowledge (DHFKS), self-care (SCHFI)	Increased knowledge and self-care
Karami (2021) [32] Iran	4 consecutive days; 20–30 min sessions; nurses	2 weeks	HF self-care scales	Teach-Back effective in improving self-care
Mesbahi (2020) [37] Iran	4 individual educational sessions	3 months	Self-care scales, readmissions	Improved self-care and reduced readmissions
Mohammadi (2021) [33] Iran	Teach-Back educational sessions during hospitalization	3 months	SF-36, Cardiac Anxiety Questionnaire	Improved QoL and reduced anxiety
Oh (2023) [29] South Korea	HEART program with pre-discharge Teach-Back	1 month	SCHFI v7.2; symptom questionnaire	Improvement in self-care
Peter (2015) [20] USA	Standardized Teach-Back + educational checklists	30 days	HF readmissions	Reduction in readmissions
Rahmani (2020) [38] Iran	Nursing education with Teach-Back assessment	3 months	HF knowledge, SF-36	Improvement in knowledge and QoL
Saadatian (2022) [22] Iran	3 consecutive sessions (30–45 min)	1 month	Brief IPQ; CMSES	Improvement in illness perception and self-efficacy
Vesterlund (2015) [21] USA	Teach-Back tool + discharge bundle	30 days	HF readmissions	Reduction in readmissions
Voelliger (2021) [23] Switzerland	Education on pulse self-assessment with Teach-Back	Short-term follow-up	Ability to recognize arrhythmia	Improvement in patient skills
White (2013) [19] USA	Discharge education with Teach-Back and follow-up phone call	7 days	HF knowledge; readmissions	Better knowledge retention
Zabolypour (2020) [39] Iran	Individual educational sessions	3 months	Treatment adherence	Both effective, Teach-Back improves adherence

Brief IPQ, Brief Illness Perception Questionnaire; CMSES, Cardiovascular Management Self-efficacy Scale; DHFKS, Dutch Heart Failure Knowledge Scale; HF, heart failure; MMAS-8, 8-item Morisky Medication Adherence Scale; QoL, quality of life; RCT, randomized controlled trial; SCHFI, Self-Care of Heart Failure Index; SF-36, 36-Item Short Form Health Survey.

**Table 4:** Cardiovascular diseases.

Study	Heart Failure	Coronary Artery Disease; Acute Coronary Syndrome; Coronary Artery Bypass Grafting	Atrial Fibrillation	Hypertension
Aghamohammadi et al. [34]	✓			
Awoke et al. [35]	✓			
Azizi et al. [36]		✓		
Bates et al. [40]		✓		
Charais et al. [41]	✓			
Dalir et al. [30]	✓			
Dinh et al. [31]	✓			
Karami Salaheddin Kola et al. [32]	✓			
Mesbahi et al. [37]	✓			
Mohammadi et al. [33]	✓			
Oh et al. [29]	✓			
Peter et al. [20]	✓			
Rahmani et al. [38]	✓			
Saadatian et al. [22]		✓		
Vesterlund et al. [21]	✓			
Voelliger et al. [23]			✓	
White et al. [19]	✓			
Zabolypour et al. [39]				✓

**Table 5:** Teach-Back providers and training.

Study	Provider	Teach-Back Training
Aghamohammadi et al., 2020 [34]	Not explicitly reported; intervention described as structured patient education likely delivered in a nursing context	Not reported
Awoke et al., 2019 [35]	Nurses (principal investigator and unit nurses)	Training described: three consecutive “lunch-and-learn” sessions
Azizi et al., 2020 [36]	Nurses in the coronary care unit (CCU)	Not reported
Bates et al., 2014 [40]	Patient educator	Preparation included review of the Health Literacy and Patient Safety clinician manual
Charais et al., 2020 [41]	Patient Care Coordinator (registered nurse); Clinical Nurse Specialist if PCC unavailable	Not reported
Dalir et al., 2015 [30]	Not explicitly reported; individual bedside education likely delivered by a nurse or nurse researcher	Not reported
Dinh et al., 2019 [31]	Nurses	Nurses reported to have received Teach-Back training, but no details provided
Karami Salaheddin Kola et al., 2021 [32]	Not specified; intervention included bedside sessions and follow-up phone calls	Not reported
Mesbahi et al., 2020 [37]	Nurses	Not reported
Mohammadi et al., 2021 [33]	Researcher (educational sessions and discussion following multimedia component)	Not reported
Oh et al., 2023 [29]	Cardiovascular unit nurse	Described as “well-trained” with 5 years of cardiovascular nursing experience; Teach-Back training not specified
Peter et al., 2015 [20]	Clinical nursing staff with multidisciplinary involvement	Training included 20 min eLearning module and mandatory 2 h “train-the-trainer” workshop
Rahmani et al., 2020 [38]	Nurses	Not reported
Saadatian et al., 2022 [22]	Nursing team	Not reported
Vesterlund et al., 2015 [21]	Staff nurses; follow-up calls performed by charge nurse, staff nurse, or nurse practitioner	Training included PowerPoint education and pre/post knowledge test (15 items) on HF education and Teach-Back strategies
Voelliger et al., 2021 [23]	Nursing staff	Not reported
White et al., 2013 [19]	Two heart failure registered nurse coordinators	Training through course offered by the Institute for Healthcare Improvement
Zabolypour et al., 2020 [39]	Not reported; Teach-Back protocol described without specifying the provider	Not reported

**Table 6:** Data collection instruments.

Study/Year	Validated Instruments	Non-Validated Measures/Questionnaires	Clinical Indices	Clinical or Administrative Data Sources
Aghamohammadi et al. [34]	MMAS-8			
Awoke et al. [35]	Dutch Heart Failure Knowledge Scale (DHFKS); Self-care for Heart Failure Index (SCHFI v6.2) Confidence and Conviction Scale			
Azizi et al. [36]		Adherence questionnaire		
Bates et al. [40]		Structured education documentation		Electronic medical record (HER)
Charais et al. [41]				Program audit/readmission data
Dalir et al. [30]	European Heart Failure Self Care Behavior (EHFScBS)			
Dinh et al. [31]	DHFKS; SCHFI v6.2		Charlson Comorbidity Index	
Karami Salaheddin Kola et al. [32]	DHFKS; EHFScBS; Multidimensional Scale of Percepioned Social Support (MSPSS)			
Mesbahi et al. [37]	European Heart Failure Self Care Behavior (EHFScBS)			
Mohammadi et al. [33]	MLHFQ; Cardiac Anxiety Questionnaire			
Oh et al. [29]	SCHFI v7.2; Korean version of Symptom Status Questionnaire-Heart Failure		Charlson Comorbidity Index	
Peter et al. [20]				Electronic medical record (EHR)
Rahmani et al. [38]	SF-36 Questionnaire; Cardiac Self-Care Questionnaire			
Saadatian et al. [22]	Brief Illness Perception Questionnaire (Brief-IPQ); Cardiovascular Management Self-Efficacy Scale (CMSES)			
Vesterlund et al. [21]		Call Back Questionnaire		Electronic medical record (EHR)
Voelliger et al. [23]		Pulse self-check assessment	CHA_2_DS_2_-VASc	
White et al. [19]				Social Security Death Index
Zabolypour et al. [39]	Hypertension adherence scale			

## Data Availability

No new data were created or analyzed in this study. Data sharing is not applicable to this article.

## Supplementary Materials

The following supporting information can be downloaded at: https://www.mdpi.com/article/10.3390/healthcare14142093/s1, Table S1: Search strategy; Table S2: Quality appraisal (Dixon-Woods et al., 2005 [28]).

## Abbreviations

The following abbreviations are used in this manuscript:

ACSC	Ambulatory Care Sensitive Conditions
Brief-IPQ	Brief Illness Perception Questionnaire
CABG	Coronary Artery Bypass Grafting
CAHPS	Consumer Assessment of Healthcare Providers and Systems
CHA_2_DS_2_-VASc	Congestive heart failure, Hypertension, Age ≥ 75 years, Diabetes mellitus, Stroke/transient ischemic attack/thromboembolism, Vascular disease, Age 65–74 years, Sex category
CINAHL	Cumulative Index to Nursing and Allied Health Literature
CMSES	Cardiovascular Management Self-Efficacy Scale
CVD	Cardiovascular diseases
DHFKS	Dutch Heart Failure Knowledge Scale
EHRs	Electronic Health Records
HF	Heart Failure
JBI	Joanna Briggs Institute
MEDLINE	Medical Literature Analysis and Retrieval System Online
MeSH	Medical Subject Headings
MSPSS	Multidimensional Scale of Perceived Social Support
NYHA	New York Heart Association
PCC	Population/Concept/Context
PRISMA-ScR	Preferred Reporting Items for Systematic reviews and Meta-Analyses extension for Scoping Reviews
RCT	Randomized Controlled Trial
SCHFI	Self-care for Heart Failure Index
STAAR	State Action on Avoidable Rehospitalizations
TB	Teach-Back

## References

[1] Cardiovascular Diseases (CVDs). https://www.who.int/news-room/fact-sheets/detail/cardiovascular-diseases-(cvds).

[2] Wang Y., Wang X., Wang C., Zhou J. (2024). Global, Regional, and National Burden of Cardiovascular Disease, 1990–2021: Results from the 2021 Global Burden of Disease Study. Cureus.

[3] Mensah G.A., Fuster V., Murray C.J.L., Roth G.A., Mensah G.A., Abate Y.H., Abbasian M., Abd-Allah F., Abdollahi A., Abdollahi M. (2023). Global Burden of Cardiovascular Diseases and Risks, 1990–2022. J. Am. Coll. Cardiol..

[4] Roth G.A., Mensah G.A., Johnson C.O., Addolorato G., Ammirati E., Baddour L.M., Barengo N.C., Beaton A.Z., Benjamin E.J., Benziger C.P. (2020). Global Burden of Cardiovascular Diseases and Risk Factors, 1990–2019. J. Am. Coll. Cardiol..

[5] Luengo-Fernandez R., Walli-Attaei M., Gray A., Torbica A., Maggioni A.P., Huculeci R., Bairami F., Aboyans V., Timmis A.D., Vardas P. (2023). Economic Burden of Cardiovascular Diseases in the European Union: A Population-Based Cost Study. Eur. Heart J..

[6] Howie-Esquivel J., Bidwell J.T. (2023). A State-of-the-Art Review of Teach-Back for Patients and Families with Heart Failure: How Far Have We Come?. J. Cardiovasc. Nurs..

[7] Samuels-Kalow M., Hardy E., Rhodes K., Mollen C. (2016). “Like a Dialogue”: Teach-Back in the Emergency Department. Patient Educ. Couns..

[8] Yen P.H., Leasure A.R. (2019). Use and Effectiveness of the Teach-Back Method in Patient Education and Health Outcomes. Fed. Pract..

[9] Batterham R.W., Hawkins M., Collins P.A., Buchbinder R., Osborne R.H. (2016). Health Literacy: Applying Current Concepts to Improve Health Services and Reduce Health Inequalities. Public Health.

[10] Geboers B., Reijneveld S.A., Koot J.A.R., De Winter A.F. (2018). Moving towards a Comprehensive Approach for Health Literacy Interventions: The Development of a Health Literacy Intervention Model. Int. J. Environ. Res. Public Health.

[11] Schapira M.M., Swartz S., Ganschow P.S., Jacobs E.A., Neuner J.M., Walker C.M., Fletcher K.E. (2017). Tailoring Educational and Behavioral Interventions to Level of Health Literacy: A Systematic Review. MDM Policy Pract..

[12] Sguanci M., Mancin S., Carù V., Simonelli N., Cangelosi G., Morales Palomares S., Ferrara G., Lo Cascio A. (2025). Exploring the Role of Advanced Practice Nurses in Cardiology: A Scoping Review. Int. Nurs. Rev..

[13] Sokos G., Kido K., Panjrath G., Benton E., Page R., Patel J., Smith P.J., Korous S., Guglin M. (2023). Multidisciplinary Care in Heart Failure Services. J. Card. Fail..

[14] Davidson P.M., Newton P.J., Tankumpuan T., Paull G., Dennison-Himmelfarb C. (2015). Multidisciplinary Management of Chronic Heart Failure: Principles and Future Trends. Clin. Ther..

[15] Shersher V., Haines T.P., Sturgiss L., Weller C., Williams C. (2021). Definitions and Use of the Teach-Back Method in Healthcare Consultations with Patients: A Systematic Review and Thematic Synthesis. Patient Educ. Couns..

[16] Talevski J., Wong Shee A., Rasmussen B., Kemp G., Beauchamp A. (2020). Teach-Back: A Systematic Review of Implementation and Impacts. PLoS ONE.

[17] Agency for Healthcare Research and Quality (2017). Implementation Quick Start Guide: Teach-Back; The Guide to Improving Patient Safety in Primary Care Settings by Engaging Patients and Families.

[18] Ha Dinh T.T., Bonner A., Clark R., Ramsbotham J., Hines S. (2016). The Effectiveness of the Teach-Back Method on Adherence and Self-Management in Health Education for People with Chronic Disease: A Systematic Review. JBI Database Syst. Rev. Implement. Rep..

[19] White M., Garbez R., Carroll M., Brinker E., Howie-Esquivel J. (2013). Is “Teach-Back” Associated with Knowledge Retention and Hospital Readmission in Hospitalized Heart Failure Patients?. J. Cardiovasc. Nurs..

[20] Peter D., Robinson P., Jordan M., Lawrence S., Casey K., Salas-Lopez D. (2015). Reducing Readmissions Using Teach-Back: Enhancing Patient and Family Education. JONA J. Nurs. Adm..

[21] Vesterlund M., Granger B., Thompson T.J., Coggin C., Oermann M.H. (2015). Tailoring Your Heart Failure Project for Success in Rural Areas. Qual. Manag. Health Care.

[22] Saadatian M., Yoosefian N., Kerman Saravi F., Yaghoubinia F. (2022). The Effect of the Teach-Back Method on Illness Perception and Self-Efficacy in Patients with Coronary Artery Disease. Med. Surg. Nurs. J..

[23] Voelliger C.M., VanderZwan K.J., Coyne E.P., Hu Y., Shammas N.W., Lisius K., King M., Lemke J.H. (2021). Education of Self-Radial Pulse Palpation and Atrial Fibrillation Signs and Symptoms. J. Community Health Nurs..

[24] Aromataris E., Lockwood C., Porritt K., Pilla B., Jordan Z. (2024). JBI Manual for Evidence Synthesis.

[25] Tricco A.C., Lillie E., Zarin W., O’Brien K.K., Colquhoun H., Levac D., Moher D., Peters M.D.J., Horsley T., Weeks L. (2018). PRISMA Extension for Scoping Reviews (PRISMA-ScR): Checklist and Explanation. Ann. Intern. Med..

[26] Page M.J., McKenzie J.E., Bossuyt P.M., Boutron I., Hoffmann T.C., Mulrow C.D., Shamseer L., Tetzlaff J.M., Akl E.A., Brennan S.E. (2021). The PRISMA 2020 Statement: An Updated Guideline for Reporting Systematic Reviews. PLoS Med..

[27] Ouzzani M., Hammady H., Fedorowicz Z., Elmagarmid A. (2016). Rayyan—A Web and Mobile App for Systematic Reviews. Syst. Rev..

[28] Dixon-Woods M., Agarwal S., Jones D., Young B., Sutton A. (2005). Synthesising Qualitative and Quantitative Evidence: A Review of Possible Methods. J. Health Serv. Res. Policy.

[29] Oh E.G., Lee J.Y., Lee H.J., Oh S. (2023). Effects of Discharge Education Using Teach-Back Methods in Patients with Heart Failure: A Randomized Controlled Trial. Int. J. Nurs. Stud..

[30] Dalir Z., Reihani Z., Mazlom S.R., Vakilian F. (2016). Effect of Training Based on Teach-Back Method on Self-Care in Patients with Heart Failure. J. Maz. Univ. Med. Sci..

[31] Dinh H.T.T., Bonner A., Ramsbotham J., Clark R. (2019). Cluster Randomized Controlled Trial Testing the Effectiveness of a Self-Management Intervention Using the Teach-Back Method for People with Heart Failure. Nurs. Health Sci..

[32] Karami Salaheddin Kola M., Jafari H., Charati J.Y., Shafipour V. (2021). Comparing the Effects of Teach-Back Method, Multimedia and Blended Training on Self-Care and Social Support in Patients with Heart Failure: A Randomized Clinical Trial. J. Educ. Health Promot..

[33] Mohammadi F., Jahromi M.S., Bijani M., Karimi S., Dehghan A. (2021). Investigating the Effect of Multimedia Education in Combination with Teach-Back Method on Quality of Life and Cardiac Anxiety in Patients with Heart Failure: A Randomized Clinical Trial. BMC Cardiovasc. Disord..

[34] Aghamohammadi M., Khatiban M., Soltanian A., Khalili Z. (2020). Comparison of the Effect of Two Teach-Back Training and Pictorial Training Methods on Medication Adherence in Heart Failure Patients. Avicenna J. Nurs. Midwifery Care.

[35] Awoke M.S., Baptiste D.-L., Davidson P., Roberts A., Dennison-Himmelfarb C. (2019). A Quasi-Experimental Study Examining a Nurse-Led Education Program to Improve Knowledge, Self-Care, and Reduce Readmission for Individuals with Heart Failure. Contemp. Nurse.

[36] Azizi H., Yosefian Miandoab N., Yaghoubinia F. (2020). Effect of Teach-Back on Treatment Adherence in Patients with Acute Coronary Syndrome: A Semi-Experimental Study. J. Maz. Univ. Med. Sci..

[37] Mesbahi H., Kermansaravi F., Kiyani F. (2020). The Effect of Teach-Back Training on Self-Care and Readmission of Patients with Heart Failure. Med. Surg. Nurs. J..

[38] Rahmani A., Vahedian-Azimi A., Sirati-Nir M., Norouzadeh R., Rozdar H., Sahebkar A. (2020). The Effect of the Teach-Back Method on Knowledge, Performance, Readmission, and Quality of Life in Heart Failure Patients. Cardiol. Res. Pract..

[39] Zabolypour S., Alishapour M., Behnammoghadam M., Abbasi Larki R., Zoladl M. (2020). A Comparison of the Effects of Teach-Back and Motivational Interviewing on the Adherence to Medical Regimen in Patients with Hypertension. Patient Prefer. Adherence.

[40] Bates O.L., O’Connor N., Dunn D., Hasenau S.M. (2014). Applying STAAR Interventions in Incremental Bundles: Improving Post-CABG Surgical Patient Care. Worldviews Evid.-Based Nurs..

[41] Charais C., Bowers M., Do O.O., Smallheer B. (2020). Implementation of a Disease Management Program in Adult Patients with Heart Failure. Prof. Case Manag..

