# Supplementary material for: Use of the Teach-Back Method in Adults with Cardiovascular Disease: A Scoping Review
Source: Healthcare (Basel). 2026 Jul 13;14(14):2093. doi: 10.3390/healthcare14142093 (PMC13410930; doi:10.3390/healthcare14142093)
Supplement: Supplementary file 1 [file healthcare-14-02093-s001.zip › healthcare-4343205-supplementary.pdf]

Use of the Teach-Back Method in Adults with Cardiovascular Disease: A Scoping Review

Supplementary Table S1. Search strategy.

**MEDLINE (PubMed)***: ("Cardiovascular Diseases"[Mesh] OR "Heart Failure"[Mesh] OR "Coronary Artery Disease"[Mesh] OR "Acute Coronary Syndrome"[Mesh] OR "Myocardial Infarction"[Mesh] OR "Atrial Fibrillation"[Mesh] OR cardiovascular[Title/Abstract] OR "cardiovascular disease*"[Title/Abstract] OR "cardiac patient*"[Title/Abstract] OR "heart failure"[Title/Abstract] OR "coronary artery disease"[Title/Abstract] OR "acute coronary syndrome"[Title/Abstract] OR "myocardial infarction"[Title/Abstract] OR "atrial fibrillation"[Title/Abstract]) AND ("Teach-Back Communication"[Mesh] OR "teach-back"[Title/Abstract] OR "teach back"[Title/Abstract]).*
**Embase:** *('cardiovascular disease'/exp OR 'heart failure'/exp OR 'coronary artery disease'/exp OR 'acute coronary syndrome'/exp OR 'myocardial infarction'/exp OR 'atrial fibrillation'/exp OR cardiovascular:ti,ab OR 'cardiovascular disease*':ti,ab OR 'cardiac patient*':ti,ab OR 'heart failure':ti,ab OR 'coronary artery disease':ti,ab OR 'acute coronary syndrome':ti,ab OR 'myocardial infarction':ti,ab OR 'atrial fibrillation':ti,ab) AND ('teach-back':ti,ab OR 'teach back':ti,ab).*
**CINAHL***: (MH "Cardiovascular Diseases+" OR MH "Heart Failure+" OR MH "Coronary Artery Disease+" OR MH "Acute Coronary Syndrome+" OR MH "Myocardial Infarction+" OR MH "Atrial Fibrillation+" OR TI cardiovascular OR AB cardiovascular OR TI "cardiovascular disease*" OR AB "cardiovascular disease*" OR TI "cardiac patient*" OR AB "cardiac patient*" OR TI "heart failure" OR AB "heart failure" OR TI "coronary artery disease" OR AB "coronary artery disease" OR TI "acute coronary syndrome" OR AB "acute coronary syndrome" OR TI "myocardial infarction" OR AB "myocardial infarction" OR TI "atrial fibrillation" OR AB "atrial fibrillation") AND (TI "teach-back" OR AB "teach-back" OR TI "teach back" OR AB "teach back").*
**Scopus:** *(TITLE-ABS-KEY("cardiovascular disease" OR cardiovascular OR "cardiac patient" OR "heart failure" OR "coronary artery disease" OR "acute coronary syndrome" OR "myocardial infarction" OR "atrial fibrillation")) AND (TITLE-ABS-KEY("teach-back" OR "teach back").*

Supplementary Table S2. Quality appraisal (Dixon-Woods et al., 2005 [28]).

Study	1	2	3	4	5	6	7	8	9	10	%
Aghamohammadi et al. [34]	Y	Y	Y	Y	Y	Y	Y	Y	Y	Y	100%
Awoke et al. [35]	Y	Y	Y	Y	Y	Y	U	Y	Y	Y	90%
Azizi et al. [36]	Y	Y	Y	Y	Y	Y	U	U	Y	Y	80%
Bates et al. [40]	Y	Y	Y	Y	Y	Y	U	U	Y	Y	80%
Charais et al. [41]	Y	Y	Y	Y	U	Y	U	U	Y	Y	70%
Dalir et al. [30]	Y	Y	Y	Y	Y	Y	Y	Y	Y	Y	100%
Dinh et al. [31]	Y	Y	Y	Y	Y	Y	Y	Y	Y	Y	100%
Karami Salaheddin Kola et al. [32]	Y	Y	Y	Y	Y	Y	Y	Y	Y	Y	100%
Mesbahi et al. [37]	Y	Y	Y	Y	Y	Y	Y	Y	Y	Y	100%
Mohammadi et al. [33]	Y	Y	Y	Y	Y	Y	Y	Y	Y	Y	100%
Oh et al. [29]	Y	Y	Y	Y	Y	Y	Y	Y	Y	Y	100%

Peter et al. [20]	**Y**	**Y**	**Y**	**Y**	**Y**	**U**	**Y**	**Y**	**Y**	**Y**	**90%**
Rahmani et al. [38]	**Y**	**Y**	**Y**	**Y**	**Y**	**Y**	**U**	**U**	**Y**	**Y**	**80%**
Saadatian et al. [22]	**Y**	**Y**	**Y**	**Y**	**Y**	**Y**	**U**	**U**	**Y**	**Y**	**80%**
Vesterlund et al. [21]	**Y**	**Y**	**Y**	**Y**	**Y**	**Y**	**Y**	**Y**	**Y**	**Y**	**100%**
Voelliger et al. [23]	**Y**	**Y**	**Y**	**Y**	**Y**	**Y**	**Y**	**Y**	**Y**	**Y**	**100%**
White et al. [19]	**Y**	**Y**	**Y**	**Y**	**Y**	**Y**	**Y**	**Y**	**Y**	**Y**	**100%**
Zabolypour et al. [39]	**Y**	**Y**	**Y**	**Y**	**Y**	**Y**	**U**	**Y**	**Y**	**Y**	**90%**

Legend: Dixon-Woods criteria (2005): 1 Clear objectives, 2 Appropriate design, 3 Setting described, 4 Adequate sample, 5 Rigorous data collection, 6 Appropriate analysis, 7 Bias/limitations discussed, 8 Ethics stated, 9 Clear results, 10 Utility/transferability.
